# Erysipelas*Read before the Brazos Valley Medical Association, at Hearne, Texas, November 15, 1899.

**Published:** 1900-01

**Authors:** M. L. Lankford

**Affiliations:** Baileyville, Texas


For Texas Medical Journal.

### Erysipelas.*

BY M. L. LANKFORD, M. D., BAILEYVILLE, TEXAS.

This affection was early known by the ancients. It occurred in epidemic in France in 1750, in Great Britain in 1800, and in the United States in 1840-45. It is an acute inflammatory disease characterized by intense heat or burning, discoloration of parts involved, accompanied by high temperature, nerve perturbation and constipation. It is usually easily controlled if the integument only is involved, and patient strong and vigorous. †It is caused by the streptococcus erysipelatus, and the constitutional disturbance is due to their presence or to presence of ptomaines in the blocd. They obtain entrance into the system through wounds and abrasions, tho' it is thought by some observers that the poison may enter the system through the lungs and alimentary canal, and in support of this theory quote cases of idiopothic erysipelas. That it is contagious is no longer a question, for we have seen numerous instances in which this fact has been demonstrated. I will relate an instance to show how easily this sometimes occurs.

Mr. S., while convalescing from a very mild attack of erysipelas, visited a brother, remaining about two hours; while there bathed his face and hands and used a towel, a few minutes later same towel was used by his brother's wife. She discovered a few minutes thereafter that he had used towel and bowl; removed and sterilized both with boiling water, however, in eight or ten days she was suddenly seized with chill and rigors and in twenty-four hours developed a spot upon side of her nose that continued to spread, invading eyelids, forehead and nearly whole of cheek, accompanied by temperature ranging from 103° to 105° F., delirium, and very marked depres-

---

*Read before the Brazos Valley Medical Association, at Hearne, Texas, November 15, 1899.

†American Text-book of Surgery, page 66.

sion throughout attack. In a second instance I was called to see a gentleman that had been suffering with a tired, indisposed feeling, chilly sensations, slight headache and impaired appetite for past twenty-four hours. While at his home was called to see a patient in confinement; the labor was normal, and terminated in about three hours; placenta was discharged spontaneously and intact, uterus promptly contracted; the following thirty-six hours lochia was normal in amount, bowels moved once, and kidneys normal, temperature normal, skin moist and patient expressed herself as feeling well enough to get up. Suddenly she was seized with hard chill, followed by high temperature and general peritonitis, lasting about two weeks. In meantime, former patient from whose bedside I had been called, developed a well marked case of erysipelas. I undoubtedly was the medium through which the erysipelatous streptococci were transmitted to parturient patient. The fact I wish to call your attention to is the transmission of the streptococci before the erysipelas is manifested either by local erythema or chill, or elevated temparature. Hygenic surroundings of parturient patient were good. I had not been exposed to anything calculated in the least to cause puerperal fever. *When erysipelas supervenes upon wounds its approach is usually denoted by an arrest of the adhesive process, by a tensive, burning sensation in the affected part, by a discharge of thin, serous matter, or an entire suppression of secretion, and by an œdematous appearance of surrounding structures; finally, the characteristic blush occurs, and diffusing itself gradually, often spreads over a considerable extent of surface. It generally occurs within forty-eight hours after injury. For mild cases, usually after moving bowels by 10 gr. dose of calomel, followed in six hours by enema or castor oil and a Seidlitz powder daily thereafter, to keep bowels open, Dovers powder to keep function of skin aroused, and local applications of olive oil, laudanum and ammonia, with quinine and iron internally, usually effect a cure in a few days. In virulent cases, we are often taxed to stop the inroad of this trouble. In sthenic cases, thorough purging with calomel is absolutely imperative, in connection with full emesis. Local applications of 120 gr. nitrate of silver to ounce of water, applied to margins of inflammation and well beyond to sound tissue.

If suppuration occur lancing in half inch multiple incisions, thoroughly evacuating the pent up secretions and pus. Internally, full doses of an opiate, of which morph., in half grain doses every four

---

*Gross, Prin. and Practice of Surgery, vol. 1.

hours or oftener if necessary, is the best, should be used to allay pain and quiet nervous system. Chloral in ten grain and bromide potassium in twenty grain doses, every three or four hours, is a useful adjuvant to morphia. If inter-muscular tissue is wasting and discharge is abundant, strapping should be applied. If temperature runs high and remains up, give Antikamnia in ten grain doses to reduce fever, stimulate heart and arouse functions of skin. Also half drachm doses of tincture chloride Ferri well diluted, every four hours, sulphate quinia in three grain doses every six hours. With proper observance of hygiene, your patient will pull through.

